# A Transformer-Based Hierarchical Variational AutoEncoder Combined Hidden Markov Model for Long Text Generation

**DOI:** 10.3390/e23101277

**Published:** 2021-09-29

**Authors:** Kun Zhao, Hongwei Ding, Kai Ye, Xiaohui Cui

**Affiliations:** Key Laboratory of Aerospace Information Security and Trusted Computing, Ministry of Education, School of Cyber Science and Engineering, Wuhan University, Wuhan 430072, China; zhaokun@whu.edu.cn (K.Z.); hwding@whu.edu.cn (H.D.); kai_ye@whu.edu.cn (K.Y.)

**Keywords:** Variational AutoEncoder, text generation, Hidden Markov Model, Transformer, latent variables

## Abstract

The Variational AutoEncoder (VAE) has made significant progress in text generation, but it focused on short text (always a sentence). Long texts consist of multiple sentences. There is a particular relationship between each sentence, especially between the latent variables that control the generation of the sentences. The relationships between these latent variables help in generating continuous and logically connected long texts. There exist very few studies on the relationships between these latent variables. We proposed a method for combining the Transformer-Based Hierarchical Variational AutoEncoder and Hidden Markov Model (HT-HVAE) to learn multiple hierarchical latent variables and their relationships. This application improves long text generation. We use a hierarchical Transformer encoder to encode the long texts in order to obtain better hierarchical information of the long text. HT-HVAE’s generation network uses HMM to learn the relationship between latent variables. We also proposed a method for calculating the perplexity for the multiple hierarchical latent variable structure. The experimental results show that our model is more effective in the dataset with strong logic, alleviates the notorious posterior collapse problem, and generates more continuous and logically connected long text.

## 1. Introduction

Natural Language Processing (NLP) has achieved great progress in these years, including text classification [1,2,3,4], text generation [5,6,7,8,9,10], and sentiment analysis [11,12,13]. At the same time, deep recurrent belief [14] and capsule networks [15,16,17] also have new challenges in the field of NLP.

Deep neural networks [18] have achieved great success in the so-called neural generation of text [5,6,7,8,9,10]. In the field of text generation, the most common is the sequence-to-sequence(seq2seq) model [19], which consists of an encoder and a decoder. The encoder encodes the text into a fixed vector, and the decoder decodes the corresponding text according to this vector. However, since the encoder in seq2seq encodes the text into a fixed vector, the text generated by the decoder is too monotonous and cannot generate diverse text. The Variational AutoEncoder (VAE) [20,21] randomly samples the encoded representation vector from the hidden space, and the decoder can generate real and novel text based on the latent variables. VAE provides a tractable method to train generative models of latent variables. In NLP, latent variables may represent higher-level meanings of sentences, such as semantics or topics. These latent variables control the generation of text [22]. Compared with the traditional seq2seq model, the latent variables in the VAE are considered to make the model more powerful.

In Natural Language Generation, Long Short Term Memory networks (LSTM) [23] and Transformer structures [24] are mostly used in the text generation field. The Transformer completely uses the attention mechanism. There are many Transformer-based models, including Bert [25], UniLM [26], BART [27], T5 [28], and GPT-2 [8]. Most VAEs use the LSTM structure for text generation [22,29,30,31], and very few VAEs use the Transformer structure [32,33,34].

At present, VAE is mainly used to generate short text (less than 20 words or one sentence) in text generation, and few people pay attention to the generation of long text. For long texts, words are often composed of sentences, and sentences are composed of text. Each sentence may contain different semantics and other latent variables information, and the latent variables of each sentence may also have a progressive relationship. However, few people focused on it. Therefore, in order to improve long text generation, we proposed a new method for combining the Transformer-Based Hierarchical Variational AutoEncoder and Hidden Markov Model (HT-HVAE) to learn multiple hierarchical latent variables and their relationships.

Humans think about their purpose before writing, which is the meaning of this article. After that, an individual will write by progressing from sentence to sentence. The act of writing these sentences needs to meet the writing purpose, and the previous sentence simultaneously determines the generation of the following sentence. However, the purpose of writing is not observable. For example, when reading a long text, we can only know the author’s writing purpose if we read the entire text. In addition, each sentence in the text has its own semantics or topics. These are also latent variables, but these latent variables, such as semantics or topics, cannot exist without the purpose of writing. It is these latent variables that control the generation of the entire long text. We call the latent variables that control the generation of all sentences similar to the purpose of writing as global latent variables, and the latent variables that specifically control the generation of each sentence (such as semantics or topics) are called local latent variables. In addition, the current local latent variables are also related to the previous local latent variables (for example, semantic conversion, etc.).

We used a Hierarchical Transformer to encode long text for the encoder of HT-HVAE. We used a word-level Transformer to encode every sentence (we denoted it as sentence-code). All sentence-code passes sentence-level transformers to acquire text representations (we denote the represent as text-code). The sentence-code will be used to learn local latent variables and text-code to learn global latent variables. The decoder also applies hierarchical structure, which combines Gated Recurrent Unit (GRU) [35] with GPT-2, to reconstruct texts. The input of GRU includes local latent variables, and we denote GRU’s output as plan-vectors. The plan-vector will be copied in n copies, where n is the length of the sentence as the input of GPT-2 to generate the sentence.

We always maximize Evidence Lower Bound (ELBO) in the training process of VAE. Thus, we can use ELBO to approximately calculate the perplexity (PPL) of the language model. However, ELBO always makes the calculated PPL inaccurate. The Importance Weighted AutoEncoder (IWAE) [36] proposed a method that PPL can calculate a single latent variable as accurately as possible. Many researchers used this method to calculate PPL as accurately as possible [33]. However, this method is mainly for single latent variables, and HT-HVAE has multiple latent variables. We adopted the idea of IWAE and expanded the method of accurately calculating PPL in the case of multi-level latent variables.

In summary, our work mainly explores the following:

1. In order to further generate real long texts, we propose a new model that combines VAE, HMM, and hierarchical structure to learn one global latent variable and multiple local latent variables.
2. The global latent variables (from the entire text) learned by our method control the local latent variables (from each sentence), and the local latent variables at the sentence level also have dependencies. Moreover, this is similar to the way humans write.
3. According to IWAE, we extend this method for the hierarchical latent variable in order to obtain a more accurate PPL.
4. Experiments prove that our model alleviates the notorious posterior collapse problem in VAE.

The topic in the first section is the Introduction. The following sections are structured as follows: Section 2 introduces related work, Section 3 introduces background knowledge, Section 4 introduces the methods we use, Section 5 introduces experimental results, and Section 6 is the conclusion.

## 2. Related Work

VAE has made significant progress in text generation [22,30,32,33,34,37,38,39,40,41,42,43]. In this reference [22], researchers used VAE for text generation for the first time. Researchers proved that learning higher-level text representations (such as semantics or topics) is possible through LSTM-based latent variables generative models, and these higher-level representations control text generation. Due to the sampling process of latent variables, the diversity of the generated text is better. [44].

However, most of VAE’s work focuses on the generation of short texts (mostly one sentence), and few people use VAE to generate long texts. Shen et al. [29] proposes a new hierarchical structure named hVAE to learn a generative model of two-layer latent variables to generate long text: the global latent variables of the entire text and the local latent variables at the sentence level. However, hVAE still has some problems: (1) There is only one sentence-level local latent variable, and it does not learn the local latent variables of each sentence. (2) It does not explore the dependency relationship between sentence-level local latent variables, for example, semantic transformation. (3) Its framework does not deviate from the LSTM structure.

Recently, the Transformer has achieved great success in the field of NLP and has surpassed LSTM, and the most famous one in text generation is GPT-2. However, the attention mechanism of GPT-2 only pays attention to the relationship between words and does not pay attention to the relationship between sentences. In addition, few people combine GPT-2 or Transformer and VAE. Liu et al. [32] combined Transformer and VAE for text generation for the first time; Wang et al. [34] used Transformer and CVAE for story complement tasks; Li et al. [33] used Bert as the encoder for VAE, and GPT-2 is used as a VAE decoder to train a large pretraining model to solve various tasks of natural language processing. However, Optimus [33] only uses the standard VAE method; that is, it only learns one hidden variable. Therefore, its application scenario is still short text, which is not suitable for long text generation. In addition, even though Optimus provides latent variable information in the decoder part, it still fails to make GPT-2 pay attention to the connection between the latent variables at the sentence level.

By considering the shortcomings of hVAE, GPT-2, and Optimus, we proposed HT-HVAE to learn a global latent variable (from the entire text) and multiple local latent variables (from each sentence). Furthermore, we propose it to learn the dependence between global latent variables and local latent variables and the connection between local latent variables. In addition, in the decoder part, we input local latent variables into GPT-2 so that GPT-2 pays attention to the relationship between words and the relationship between sentence-level latent variables.

However, text VAE also has a notorious posterior collapse problem. In recent years, several methods have been working to alleviate this problem, including different Kullback–Leibler (KL) annealing/thresholding schemes [22,45,46,47], decoder architectures [38,48], auxiliary loss [44], semi amortized inference [41], aggressive encoder training schedule [49], and flexible posterior [50]. However, none of them help to improve the perplexity compared to a plain neural language model. In this article, because the local latent variable sent to the decoder not only contains the information of the global latent variable but also contains the information of the previous local latent variable, it makes the prior distribution function of the local latent variable more flexible. Therefore, the decoder contains more local latent variable information, which alleviates the problem of posterior collapse.

## 3. Background

### 3.1. VAE

**Figure 1:**
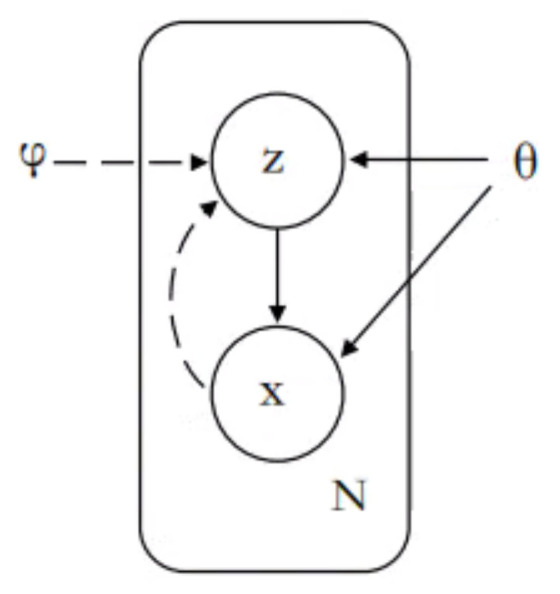
VAE. The solid line represents the generative network, and the dashed line represents the inference network [20].

As shown in Figure 1, VAE mainly includes inference networks and generative networks. In NLP, the inference network mainly represents sentences as low-dimensional latent variables, and the generative network uses this latent variable to generate sentences. In fact, VAE can be regarded as a regularized version of the AutoEncoder (AE) [18], but the low dimension of the sentence is not a fixed code, unlike AE, and is sampled from a probability distribution.

Suppose *x* is the observable data obtained by sampling, such as a sentence, and *z* is a low-dimensional latent variable, such as semantics or topics. The joint probability density function of this generative model can be decomposed into the following.

(1) p(x,z|θ)=p(z|θ)p(x|z;θ)

p(z|θ) is the prior distribution of the latent variable *z*, p(x|z;θ) is the conditional probability distribution of *x* when *z* is known, and θ is the parameter of the two probability distributions. We assume that p(z|θ) and p(x|z;θ) are a known distribution family, such as a normal distribution. In VAE, we used two neural networks to fit these two distribution families separately. In fact, we assume the standard normal distribution of p(z) and use a network to learn the probability distribution p(x|z;θ). This network is called the generative network, also called decoder, and its input is a latent variable *z* while the output is the observable variable *x*.

The purpose of the inference network is to learn the probability distribution q(z|x;ϕ), which is used to approximate the true posterior distribution p(z|x;θ). The input of the inference network is *x*, and the output is the probability distribution q(z|x,θ). Similarly, we assume that q(z|x;θ) is Gaussian distribution.

It has been proved in [20] that the objective function of VAE is ELBO, which is composed of the reconstruction term and the KL divergency.

(2) $$ELBO=E_{q(z|x;\phi )}logp\left(x|z;\theta \right)-KL\left(q\left(z|x;\theta \right)||p\left(z;\theta \right)\right)$$

The first term in ELBO is called the reconstruction loss, which is the same as the AE, and the second term is KL loss, which can be regarded as a regularized item. The KL loss forces the distribution of the hidden variable *z* to approximate p(z;θ) as much as possible and to sample from it in order to generate real text.

When VAE is used in the field of text generation, most of the inference network and the generative network utilize the LSTM structure. Due to the strong decoding ability of LSTM, *x* can be generated without *z* during the generation process, so the notorious “posterior collapse” problem will occur. In HT-HVAE, we use Transformer as the inference network and the generative network. For single-layer latent variables, the problem of posterior collapse will also occur. However, we have learned two layers of latent variables, namely the global latent variable $z_{t}$ of the entire text and the local latent variable $z_{i}$ of each sentence.

Moreover, for each sentence, the prior latent variables $z_{i}$ are related to prior hidden variables $z_{i-1}$ of the previous sentence. The prior distribution of $z_{i}$ is no longer a simple standard diagonal Gaussian distribution, rendering the local latent variable $z_{i}$ more important when decoding. Experiments show that HT-HVAE alleviates the problem of posterior collapse.

### 3.2. Transformer

The Transformer [24] is a new sequence-to-sequence model, different from Convolutional Neural Networks (CNNs) and Recurrent Neural Networks (RNNs), and it only relies on the attention mechanism. Since its inception, Transformer has achieved great success in many areas of natural languages, such as machine translation and other natural language understanding tasks.

**Figure 2:**
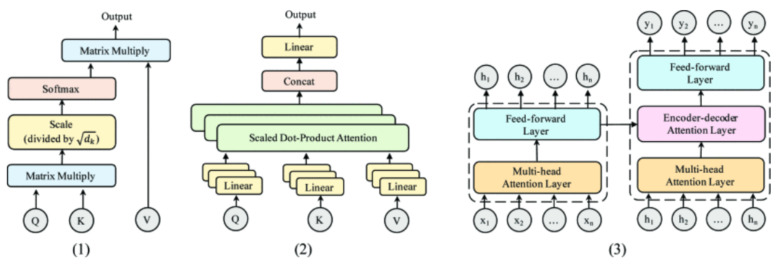
Transformer. (1) Scaled Dot-Product Attention, (2) Multi-Head Attention, and (3) Transformer model [24].

The multi-head self-attention mechanism is the core of the Transformer. It mainly consists of two parts: Scaled Dot-Product Attention and Multi-Head Attention. Generally speaking, the point of Scaled Dot-Product Attention is to map a query and a key-value into an output, as shown in (1) of Figure 2. This output is the weighted sum of key values, and its weight is calculated from the similarity between query and key. In the self-attention mechanism, the similarity between query and key is scaled dot-product. Its form is as follows.

(3) $$Attention\left(Q,K,V\right)=softmax\left(\frac{QK^{T}}{\sqrt{d_{k}}}\right)V$$

*Q*, *K*, and *V* are very common in the attention mechanism. They represent Query vector, Key vector, and Value vector, respectively, and they are all learnable parameters. By using this method, Transformer can learn the distribution of attention between words. Moreover, in order to retain a smaller inner product of *Q* and *K*, it is usually divided by the dimension of *K*.

In addition, Transformer uses a method called multi-head self-attention mechanism, shown in the (2) of Figure 2. First, different linear functions are used to divide *Q*, *K*, and *V* into different h parts. Each part’s self-attention score is then calculated, and these h parts are then concatenated through the linear function as the final output:

(4) $$MultiHead\left(Q,K,V\right)=Concat\left(head_{1},\ldots ,head_{h}\right)$$

(5) $$wherehead_{i}=Attention\left(QW_{i}^{Q},KW_{i}^{K},VW_{i}^{V}\right)$$

where the matrices $QW_{i}^{Q}\in R^{d_{model}*d_{k}}$, $KW_{i}^{K}\in R^{d_{model}*d_{k}}$, and $VW_{i}^{V}\in R^{d_{model}*d_{k}}$ represent the corresponding linear projections.

(3) In Figure 2, the entire structure of the Transformer is presented: The left one is the encoder, and the right one is the decoder. In particular, the Transformer’s encoder consists of a multi-head self-attention layer and a feed-forward fully connected layer. Such modules have *N* layers in total, and each layer’s module accepts the output of the previous layer as the input of the layer. The encoder’s function is to encode the input *x* into H and then to send *H* to the decoder.

The decoder is similar to the encoder. The only difference is that an encoder–decoder multi-head self-attention layer is added. An encoder–decoder attention layer is directly added to the multi-head attention layer and the feed-forward fully connected layer. There are also *N* such modules. The encoder–decoder multi-head self-attention layer’s query is the output of the previous decoder layer, and the key and value come from the output of the encoder.

### 3.3. HMM

The Hidden Markov Model (HMM) is a relatively classic machine learning model. It is widely used in language recognition, natural language processing, pattern recognition, and other fields.

Of course, with the current rise of deep learning, especially the popularity of neural network sequence models such as RNN and LSTM, the status of HMM has declined.

**Figure 3:**
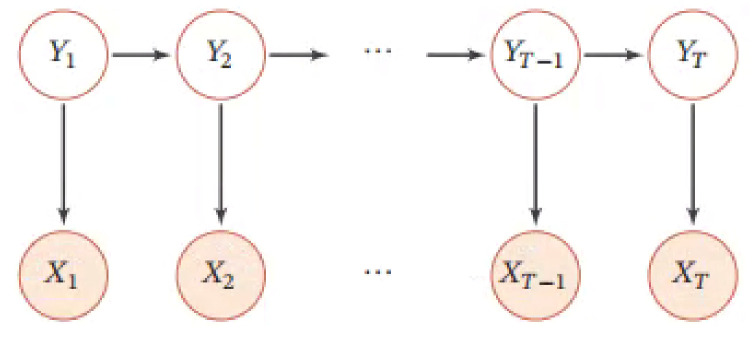
HMM. $X_{1:T}=(X_{1},X_{2},\;$…$,X_{T})$ represents an observable variable, and $Y_{1:T}=(Y_{1},Y_{2},\;$…$,Y_{T})$ represents a hidden variable.

Figure 3 shows the probability graph model of HMM, where $X_{1:T}=(X_{1},X_{2},\;$…$,X_{T})$ represents an observable variable and $Y_{1:T}=(Y_{1},Y_{2},\;$…$,Y_{T})$ represents a hidden variable. The latent variables form a Markov chain, and each observable variable $x_{t}$ depends on the current latent variable $y_{t}$, and its joint probability distribution is the following.

(6) $$p\left(x,y;\theta \right)=\prod_{i=0}^{n}p\left(y_{t}|y_{t-1},\theta_{s}\right)p\left(x_{t}|y_{t},\theta_{t}\right)$$

x and y, respectively, represent observable variables and hidden variables, $p(x_{t}|y_{t},\theta_{t})$ represents the output probability, $p(y_{t}|y_{t-1},\theta_{s})$ represents the transition probability, and $\theta_{t}$ and $\theta_{s}$ represent the parameters of both.

## 4. Method

### 4.1. Graphical Model of HT-HVAE

**Figure 4:**
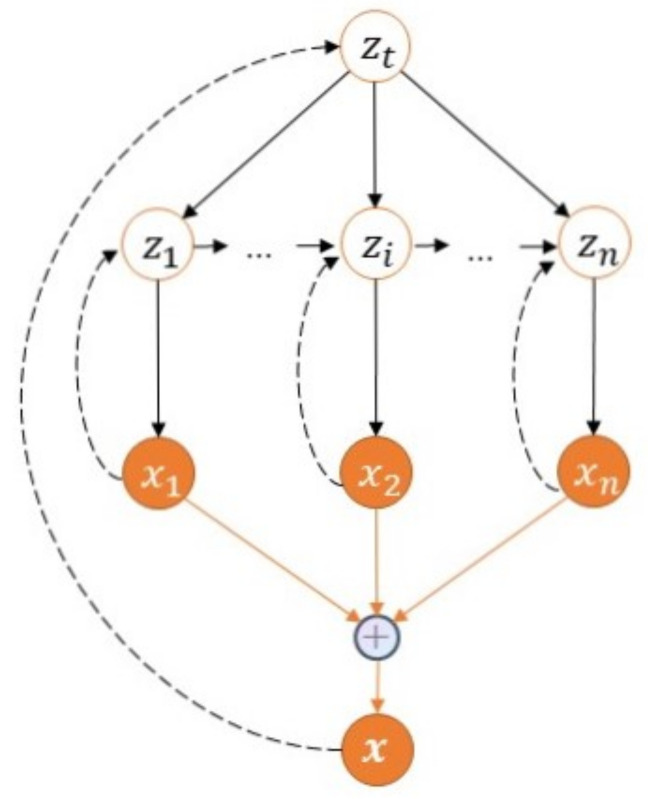
Graphical Model of HT-HVAE. $z_{t}$ represents the global latent variable, and $z_{i}$ represents the local latent variable. We assume that the global latent variable determines the local hidden variable, *x* represents the text data, the solid line represents the generative network, and the dashed line represents the inference network.

Unlike the previous VAE method that only captures one latent variable, our proposed HT-HVAE captures one global latent variable (from the entire text) and multiple local latent variables (from each sentence). The global latent variable controls the local latent variables, and the following local latent variables also have a dependency on the previous local latent variables at the same time. It is in line with the human writing habits mentioned in the Introduction of the article, that is, the semantics of the entire text or the purpose of writing controls the semantics of each sentence, and there is also a semantic conversion relationship between sentences. Figure 4 shows the Graphical Model of our method.

According to the Graphical Model, the joint probability density of the generative model is the following.

(7) $$p\left(x,z,z_{t};\theta \right)=p\left(z_{t}\right)\prod_{i=1}^{n}p\left(z_{i}|z_{i-1},z_{t};\theta_{1i}\right)p\left(x_{i}|z_{i};\theta_{2i}\right)$$

$x=(x_{1},\;$…$,x_{i},\;$…$,x_{n})$ and $z=(z_{1},\;$…$,z_{i},\;$…$,z_{n})$ are local latent variables, $z_{t}$ represents global latent variable, and we assume that $p(z_{t})$ possesses standard normal distribution; thus, it has no parameters. $\theta =(\theta_{1i},\theta_{2i}$) are parameters. $\theta_{1i}$ is the parameter of the prior probability distribution function of $z_{i}$, while $\theta_{2i}$ is the parameter of generative model.

Suppose that, in the inference network, $z_{t}$ and z are conditionally independent given the input x, and $z_{i}$ is also conditionally independent given the input $x_{i}$, which is described as follows.

(8) $$q\left(z_{t},z|x;\phi \right)=q\left(z_{t}|x;\phi_{t}\right)\prod_{i=1}^{n}q\left(z_{i}|x_{i};\phi_{i}\right)$$

$\phi =(\phi_{t},\phi_{i})$ are parameters. In the generative network, given that the local latent variable $z_{i}$ is determined by the global latent variable $z_{t}$ and the previous local latent variable $z_{i-1}$, we have the following.

(9) $$p\left(z_{t},z;\theta_{1}\right)=p\left(z_{t}\right)\prod_{i=1}^{n}p\left(z_{i}|z_{t},z_{i-1};\theta_{1i}\right)$$

Under the assumptions of (8) and (9), the ELBO yields the following.

(10) $$\begin{array}{cc} logp(x) & =log\int_{z_{t}}\int_{z}p\left(x,z,z_{t}\right)=logE_{q(z_{t},z|x)}\frac{p\left(z_{t}\right)\prod_{i=1}^{n}p\left(z_{i}|z_{i-1},z_{t}\right)p\left(x_{i}|z_{i}\right)}{q\left(z_{t}|x\right)\prod_{i=1}^{n}q\left(z_{i}|x_{i}\right)}\\ & \geq E_{q(z_{t},z|x)}log\frac{p\left(z_{t}\right)\prod_{i=1}^{n}p\left(z_{i}|z_{i-1},z_{t}\right)p\left(x_{i}|z_{i}\right)}{q\left(z_{t}|x\right)\prod_{i=1}^{n}q\left(z_{i}|x_{i}\right)}\\ & =\sum_{i=1}^{n}E_{q(z_{i}|x_{i})}logp\left(x_{i}|z_{i}\right)-D_{KL}(q\left(z_{t},z|x\right)||p\left(z_{t},z\right))\\ & =\sum_{i=1}^{n}E_{q(z_{i}|x_{i})}logp\left(x_{i}|z_{i}\right)-E_{q(z_{t}|x)}log\frac{q(z_{t}|x)}{p(z_{t})}\\ & -\sum_{i=1}^{n}E_{q(z_{t},z|x)}log\frac{q(z_{i}|x_{i})}{p(z_{i}|z_{i-1},z_{t})} \end{array}$$

If we create the following:

(11) $$\begin{array}{cc} I & =E_{q(z_{t},z|x)}log\frac{q(z_{i}|x_{i})}{p(z_{i}|z_{i-1},z_{t})}=\int_{z_{t}}\int_{z}q\left(z_{t}|x\right)\prod_{i=1}^{n}q\left(z_{i}|x_{i}\right)log\frac{q(z_{i}|x_{i})}{p(z_{i}|z_{i-1},z_{t})}\\ & =\int_{z_{t}}\int_{z_{i-1}}q\left(z_{t}|x\right)q\left(z_{i-1}|x_{i-1}\right)q\left(z_{i}|x_{i}\right)log\frac{q(z_{i}|x_{i})}{p(z_{i}|z_{i-1},z_{t})}\\ & =E_{q(z_{t}|x)}E_{q(z_{i-1}|x_{i-1})}q\left(z_{i}|x_{i}\right)log\frac{q(z_{i}|x_{i})}{p(z_{i}|z_{i-1},z_{t})}\\ & =E_{q(z_{t}|x)}E_{q(z_{i-1}|x_{i-1})}D_{KL}(q\left(z_{i}|x_{i}\right)||p\left(z_{i}|z_{i-1},z_{t}\right)) \end{array}$$

we, thus, obtain the following.

(12) $$\begin{array}{cc} L_{HT-HVAE} & =ELBO=\sum_{i=1}^{n}E_{q(z_{i}|x_{i};\phi_{i})}logp\left(x_{i}|z_{i};\theta_{2i}\right)-D_{KL}(q\left(z_{t}|x;\phi_{t}\right)||p\left(z_{t}\right))\\ & -\sum_{i=1}^{n}E_{q(z_{t}|x;\phi_{t})}E_{q(z_{i-1}|x_{i-1};\phi_{i-1})}D_{KL}(q\left(z_{i}|x_{i};\phi_{i}\right)||p\left(z_{i}|z_{i-1},z_{t};\theta_{1,i-1}\right)) \end{array}$$

We assume that both the prior distribution and the posterior distribution are in the form of Gaussian distributions; two KL divergences can then be obtained for closed-form solutions.

We deleted HMM from HT-HVAE to prove the effectiveness of HMM. The encoder and decoder are the same as those in our model, but the exclusion of HMM, which is the prior distribution of $z_{i}$, is only determined by $z_{t}$. The difference from hVAE is that each sentence will learn $q(z_{i}|x_{i})$ and sample from it during training. The ELBO becomes the following.

(13) $$\begin{array}{cc} L_{HT-VAE} & =ELBO=\sum_{i=1}^{n}E_{q(z_{i}|x_{i};\phi_{i})}logp\left(x_{i}|z_{i};\theta_{2i}\right)-D_{KL}(q\left(z_{t}|x;\phi_{t}\right)||p\left(z_{t}\right))\\ & -\sum_{i=1}^{n}E_{q(z_{t}|x;\phi_{t})}D_{KL}(q\left(z_{i}|x_{i};\phi_{i}\right)||p\left(z_{i}|z_{t};\theta_{1i}\right)) \end{array}$$

We hereby name it HT-VAE.

### 4.2. Model Architecture

**Figure 5:**
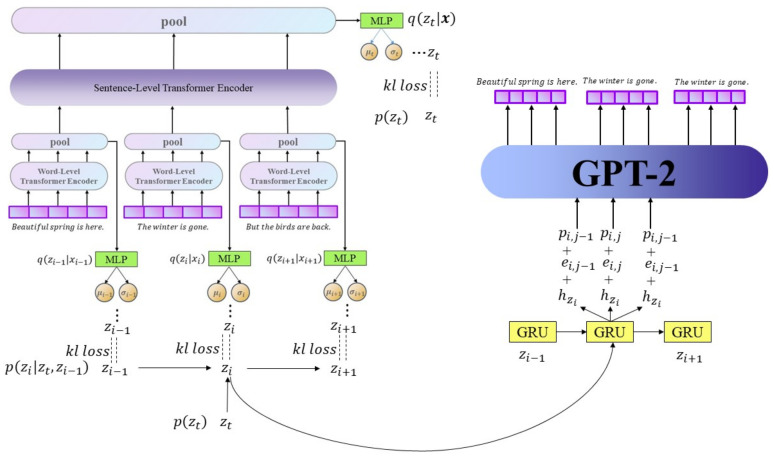
Model Architecture.The left side represents the inference network, and the right side represents the generative network.

We introduced the probability graph form of the model and derived ELBO in the previous section. This section introduces the specific architecture of the model. The Model Architecture is shown in Figure 5.

#### 4.2.1. Inference Network

The inference network is also called the encoder, and its main function is to fit q(z|x) through a neural network. As mentioned previously, the HT-HVAE contains a global latent variable and multiple local latent variables; thus, we use two layers of Transformer to encode words and sentences separately and learn $q(z_{i}|x_{i})$ and $q(z_{t}|x)$.

Suppose that the text *x* has n sentences, and each sentence has *s* words. We use the word-level Transformer to encode each sentence and the Transformer to encode each sentence sharing parameters. The output of the word-level Transformer is $H^{w}=(H_{0}^{w},\;$…$,H_{i}^{w},\;$…$,H_{s}^{w})$. We take $H_{0}^{w}$ as the wave code of the sentence and input the $H_{0}^{w}$ obtained from each sentence plus the position code into the sentence-level Transformer to obtain the code $H^{s}=(H_{0}^{s},\;$…$,H_{i}^{s},\;$…$,H_{n}^{s})$ of the entire text and take $H_{0}^{s}$ as the text coding.

(14) $$H_{i}^{w}=Transformer_{word}\left(x_{i}\right),1\leq i\leq n$$

(15) $$H^{s}=Transformer_{sen}\left(H_{i}^{w}\right),1\leq i\leq n$$

The advantage of using hierarchical Transformer coding is that it can learn the attention of words and words within a sentence and the attention between sentences. For long texts, there are often semantic relations between sentences. The experiments also proved that the PPL of hierarchical coding is lower than that of non-hierarchical coding.

After obtaining the representation of each sentence $H_{i}^{w}$ and the representation of the entire text $H^{s}$, we used a three-layer MLP network to learn the local posterior distribution and the global posterior distribution.

(16) $$\mu_{i}^{{}^{\prime }},\sigma_{i}^{2^{\prime }}=MLP\left(H_{i}^{w}\right)$$

(17) $$\mu_{t}^{{}^{\prime }},\sigma_{t}^{2^{\prime }}=MLP\left(H^{s}\right)$$

$\mu_{i}^{{}^{\prime }}$ and $\sigma_{i}^{2^{\prime }}$ are the mean and variance of $q(z_{i}|x_{i})$. $\mu_{t}^{{}^{\prime }}$ and $\sigma_{t}^{2^{\prime }}$ are the mean and variance of $q(z_{t}|x)$. We have to learn multiple local posterior distributions, and these MLPs that learn the local posterior distribution share parameters.

#### 4.2.2. Generative Network

In the generative network, the decoder is used to estimate p(x|z). Its input is a hidden variable *z*, and its output is text *x*. In the standard VAE, *z* is taken from q(z|x) when training, and *z* is taken from the prior distribution p(z) when generating text. In our model, the same is true. However, the difference is that the $z_{i}$ generated by each sentence is taken from a different $q(z_{i}|x_{i})$ during training, and $z_{i}$ is taken from a different $p(z_{i}|z_{t},z_{i-1})$ during generating.

The ELBO is mentioned in the third section, and we need to calculate KL divergences. We assume that $p(z_{t})$ is the standard Gaussian distribution, $D_{KL}(q\left(z_{t}|x\right)||p\left(z_{t}\right))$ can obtain a numerical solution. However, $p(z_{i}|z_{t},z_{i-1})$ is temporarily unknown, and another KL divergence cannot be obtained; thus, we need the MLP network to estimate $p(z_{i}|z_{t},z_{i-1})$. $z_{t}$, and $z_{i-1}$ is the vector that is used in the reparametrization trick to sample from $q(z_{t}|x)$ and $q(z_{i-1}|x_{i-1})$.

(18) $$\mu_{i},\sigma_{i}^{2}=MLP\left(z_{t},z_{i-1}\right)$$

$\mu_{i}$ and $\sigma_{i}$ are the mean and variance of $p(z_{i}|z_{t},z_{i-1})$. At this time, all KL terms can be found to be closed-form solutions. Next, we discuss the reconstruction item, which is also the most important part of the decoder.

We also use a hierarchical structure in the generative network. In the sentence-level decoder, we use the GRU network. The input of each GRU is $z_{i}$ sampled from the posterior distribution $q(z_{i}|x_{i})$ of each sentence, and what we call the plan vector $h_{i}$ is generated. Next, $h_{i}$ will be copied into s parts (s is the length of each sentence), which is used as word-level input along with word embedding and position coding.

(19) $$h_{i}=GRU\left(z_{i}\right)$$

(20) $$G_{in}=e_{ij}+h_{ij}+p_{ij}$$

$e_{ij}$ represents the word embedding of the jth word of the i-th sentence in the text, and $p_{ij}$ is the position code, $h_{ij}=h_{i}$.

We used 117M pretrained GPT-2 and $G_{in}$ as the input of GPT-2 in order to obtain $H_{in}$. Then, it will proceed through the linear layer in order to obtain the final logits. We will add $h_{i}$ and $H_{in}$ as the input of the linear layer to further strengthen the application of hidden variables in the word-level decoder.

## 5. Experiment

Our experiment mainly includes unconditional long text generation and conditional long text generation. For unconditional text generation, we used the Arxiv paper abstract dataset and Yelp comment dataset; for conditional generation, we used the Arxiv paper abstract dataset. For the evaluation of the model, we mainly evaluated the language model and the generated text. Since our paper mainly studies VAE for the generation of long texts, we only consider similar models. We use hVAE [29], GPT-2 [8], and OPTIMUS [33] as the benchmarks for language models; for the evaluation of generated text, we use ARAE [51], hVAE, GPT-2, and OPTIMUS as benchmarks.

### 5.1. Dataset

We used the Arxiv paper abstract dataset and Yelp review dataset to verify the effectiveness of our model. Arxiv paper abstracts are more logical, while Yelp comments are not as logical as Arxiv paper abstracts.

We have performed some processing on the two datasets as we are concerned about the generation of long text: (1) First, use the GPT2 Tokinzer to segment each data text, calculate the number of characters, and select the number of characters between 150 and 300. The average number of characters in the long text is about 200 (much greater than the number of characters in hVAE and Optimus). (2) Use nltk to segment the text for the selected original text data and insert ’<BOS>’ at the beginning of the first sentence, insert ’<EOS>’ at the end of each sentence, and insert ’<|endoftext|>’ at the end of the last sentence. These inserted characters are not used during encoding and are instead used in the decoding stage. (3) We selected 20 million data texts as the training set and 30,000 texts as the test set. From Table 1, we can observe the specific information of the dataset. Both datasets are divided into a training set of 200,000 long text data and a test set of 30,000 long text data. At the same time, the average number of words in Arxiv is 211, the average number of characters in Yelp is 206, and the average number of sentences in the two datasets is 6.

### 5.2. Language Model

**hVAE:** The encoder uses hierarchical CNN encoding, and the decoder uses a hierarchical LSTM network to learn two layers of hidden variables, but there is only one local hidden variable.

**GPT-2:** Composed of Transformer decoder, we use a model containing 117M parameters here.

**OPTIMUS:** The encoder is composed of Bert, and the decoder is composed of GPT-2.

This section mainly compares PPL and KL on the Arxiv dataset and Yelp dataset.

When evaluating the language model, we randomly selected 3000 pieces of data from the test set and chose their average PPL and KL as the final result.

However, in the VAE training process, the optimization function we chose was ELBO, which is less than or equal to logp(x), and logp(x) cannot be completely obtained. Therefore, PPL can only be approximated in VAE. Here, we used the IWAE method to solve OPTIMUS logp(x) as accurately as possible and drew on the idea of IWAE in order to derive a method to solve the PPL of HT-HVAE with two layers of latent variables as accurately as possible:

(21) $$\begin{array}{cc} logp(x) & =logE_{\left(z_{t},z\right)\sim q\left(z_{t},z|x;\phi \right)}\frac{1}{k}\sum_{k=1}^{n}M_{k}\\ & \geq \frac{1}{k}logE_{\left(z_{t},z\right)∼q\left(z_{t},z|x;\phi \right)}\sum_{k=1}^{n}M_{k}=L_{k} \end{array}$$

where $M_{k}=\frac{p(x,z_{k},z_{t,k};\theta )}{q(z_{t,k},z_{k}|x;\phi )}$. According to IWAE, $L_{k}$ converges to logp(x) as k → *∞*. We need to decompose $p(x,z_{k},z_{t,k};\theta )$ and $q(z_{t,k},z_{k}|x;\phi )$ to obtain PPL.

$q(z_{t,k},z_{k}|x;\phi )$, where $z_{t}$ and $z_{i}$ are independent of each other, then $z_{t}$ and $z_{i}$ can be sampled in $q(z_{t}|x)$ and $q(z_{i}|x_{i})$.

We can observe from Table 2 that the PPL of HT-HVAE is three points lower than that of OPTIMUS, and KL divergence is also improved. If we delete HMM from our model, we will find that the KL divergence is lower than the model with HMM, and PPL is not as good. This shows that our model is better than OPTIMUS in the long text language model, which also proves that our previous assumptions are correct. That is, the latent variable information obtained from the long text as a whole cannot contain the latent variable information of each sentence, and there is a relationship between the hidden variables of each sentence. Similarly to people writing articles, there is always an outline first, and then sentences are written with one sentence after another. Finally, a long text is formed. In our model, $z_{t}$ plays the role of outline, $z_{i}$ is the latent variable that generates each sentence, and the relationship between sentences is expressed by HMM, which is similar to people’s writing habits.

We calculate the NLL loss of GPT-2, OPTIMUS, HT-VAE, and HT-HVAE on two datasets in order to further illustrate the effectiveness of HMM in decoding. It can be observed from Table 3 that the NLL loss of the HT-HVAE model is lower than that of other Transformer types, which shows that adding HMM is helpful for language modeling. In particular, it works better on the more logical Arxiv paper abstract data set.

### 5.3. Evaluation of the Generated Text

We used BLEU [52] to evaluate the quality of the generated text and self-BLEU to evaluate the diversity of the generated text. BLEU was proposed by IBM in 2002 for the evaluation of machine translation tasks. Its overall idea is accuracy. If a reference is given, the sentence generated by the neural network is a candidate, the sentence length is n, and m words in the candidate appear in the reference. We used this method to generate 1000 sentences as candidates and used all test set data (30,000 items) as references to calculate BLEU-2, BLEU-3, and BLEU-4, respectively.

It can be observed from Table 4 that our model surpassed the original model in B-2, B-3, and B-4 scores, reaching the state of art. This shows that our model can produce better quality text. We found that in a dataset with strong logic, the BLEU score with HMM is better than the score without HMM; on the contrary, the results of the two are similar.

We use self-BLEU [53] to evaluate the diversity of the generated text. The lower self-BLEU is, the better text diversity is. Similarly, we randomly generated 1000 yelp comments in order to evaluate the diversity of the text. It can be observed from Table 5 that our model may not be the best at the B-2 level but surpassed the previous model at the B-3 and B-4 levels. This shows that our model generates better text diversity.

The effect of our model on the yelp dataset is not much different from the baseline model, but it performs better on the Arxiv paper abstract dataset. A possible reason for this is that the corpus of Arxiv paper abstracts is richer than the Yelp dataset, and the abstract is written carefully and logically, so it is more in line with the ideas of our model. In contrast, the corpus of the Yelp comment dataset is not very rich, and the logic is definitely not as good as the Arxiv paper abstract, so our model is not much improved compared to the baseline model. The reason why our model generates better corpus diversity is due to the fact that the hidden variables that control the generation of each sentence are constantly changing, unlike other baseline models that do not change.

### 5.4. Interpolating Latent Space

According to reference [22], we measured the continuity of the learned latent variable space. First, we randomly sampled two points (denoted as $z_{tA}$ and $z_{tB}$) from the space of the prior hidden variable $z_{t}$, and then we sent them to the decoder to generate text based on the equidistant intermediate points along the linear trajectory between $z_{tA}$ and $z_{tB}$, that is, the hidden variable input into the decoder is $z_{t}=a*z_{tA}+\left(1-a\right)*z_{tB},0\leq a\leq 1$. As shown in Table 6, these intermediate samples are all realistic looking reviews that are syntactically and semantically reasonable, demonstrating the smoothness of the learned VAE latent space.

### 5.5. Conditional Text Generation

We used the Arxiv paper abstract dataset in order to achieve controllable text generation. While encoding each word, the title of the paper is also encoded but not sent to the sentence-level encoder. At the same time, we also changed the prior probability of $z_{t}$, and it is no longer the standard Gaussian distribution but the Gaussian distribution determined by the title code *c*. In the generation stage, the word of the title will also be added before ‘<BOS>’, but this part of the title will not add hidden variables unlike the sentence of the text; that is, this part of the hidden variable is zero. Given the title, the generated text is shown in Table 7.

## 6. Conclusions

In this paper, we propose a new method combining VAE+HMM+Hierarchical Transformers to generate better long texts. Unlike traditional VAE, our model learns more than one hidden variable including the global hidden variable and multiple local hidden variables. In addition, the global hidden variable and the previous local hidden variable control the current local hidden variable. Another advantage of our model is that the VAE and Transformer are further combined, and each local latent variable is sent to the decoder GPT-2 so that GPT-2 notices the connection between the local latent variables at the sentence level. This is similar to human writing habits. Experimental results prove that our model generates high-quality long texts.

However, our method still has some shortcomings. We only proposed that there are some connections between the global hidden variables and local hidden variables of the long text, which is also proved by the comparison experiment results. However, we did not conduct a quantitative analysis of the relationship between this hidden variable and analyze the strength of the relationship between them. The question of how to quantitatively analyze the relationship between such hidden variables is a difficult one to answer. This is exactly what we aim to study next.

## Figures and Tables

**Table 1:** Dataset. “Ave Toks” is average number of words, and Ave Sents” is the average number of sentences.

	Train	Test	Ave Toks	Ave Sents
Arxiv	200,000	30,000	211	6
Yelp	200,000	30,000	206	6

**Table 2:** PPL and KL.

	Arxiv		Yelp	
Model	KL	PPL	KL	PPL
hVAE	12.7	54.3	6.8	45.8
GPT-2	-	26.95	-	31.45
OPTIMUS	14.48	25.71	14.50	26.91
HT-VAE	18.14	24.31	19.78	26.28
HT-HVAE	**19.00**	**22.70**	**21.83**	**25.14**

**Table 3:** NLL loss.

	GPT-2	OPTIMUS	HT-VAE	HT-HVAE
Arxiv	695.56	686.98	668.27	**657.99**
Yelp	703.19	670.02	669.66	**667.73**

**Table 4:** BLEU.

	Yelp			Arxiv		
Model	B-2	B-3	B-4	B-2	B-3	B-4
hVAE	0.912	0.755	0.549	0.825	0.657	0.460
GPT-2	0.961	0.876	0.639	0.925	0.782	0.537
OPTIMUS	0.961	0.918	0.829	0.903	0.801	0.645
HT-VAE	0.972	**0.930**	**0.837**	0.911	0.809	0.651
HT-HVAE	**0.972**	0.929	0.833	**0.938**	**0.851**	**0.717**

**Table 5:** Self-BLEU.

	B-2	B-3	B-4
ARAE	0.725	0.544	0.402
hVAE	0.851	0.723	0.729
GPT-2	0.871	0.616	0.326
OPTIMUS	0.773	0.527	0.316
HT-VAE	0.769	0.513	0.294
HT-HVAE	0.763	**0.500**	**0.281**

**Table 6:** Interpolating latent space. The decimal on the left represents the value of *a* and the generated text on the right.

0.0	My family and I went here for dinner on a Saturday night. We had a large group and were seated right away. We had a lot of fun with the food and the service. We had the “Ribs” fried rice. The rice was great! The shrimp and grits were really good and the red potatoes were nice and crispy. The wine selection was good. The atmosphere was nice and the food was great. The service was good and the food was great. We tried the “Fajita” chicken wings. I would recommend this place to anyone in the area. The wings were great and were well seasoned. They were very flavorful and the portions were huge. The bill was around $20. I was also impressed with the friendly staff. I will be back!
0.1	I’m a fan of their food and I love their beer selection. I tried their House of Beer and it’s something I would have enjoyed if I could. They have a selection of beers and their beers are always pretty good. I’ve also tried their brunch buffet and there is a huge selection. My only problem is that they don’t have a lot of seating. The staff is super nice and they are always very courteous to all the guests. If you’re in the area and need to park, head over there and check this place out.
0.2	The restaurant is great. Our server was amazing. He’s very friendly and polite.We’ve had a few reservations and it’s always a good time. I also got the same server that I had last night. He’s very friendly and helpful. The only reason I have not come back is because of the price. I paid $5 for a side of oysters and no oysters. We have a group of 8 people and the food is pretty good. We also ordered 2 sides and 2 appetizers. They’re amazing. I’m not a huge fan of salads, but if you have salads and/or a lot of veggies, they will make it delicious. I have had more than one salad in my entire life and they are the best salad I’ve ever had. I’m not a fan of small portions, but if you have small portions, you will love it. I always have ordered the chicken and oyster and the rolls are delicious. I’ll come back here again.
0.3	I have been here a couple of times. They are very busy and usually only have to wait a couple of hours. When I go in there is a selection of fresh, homemade, and locally made tacos. If you are craving Mexican food, I suggest the smoked steak taco (sigh). I had the goat cheese taco which is very delicious. The beef in the goat cheese was tender and juicy. I also got the chipotle chips which were nice and crunchy. The tapas were a bit dry and I’m still not sure how they were prepared. My boyfriend got the guacamole taco which was amazing.It had a really good guacamole flavor to it. It was also a little cold for me. But overall, the service was great. They have a small patio area with a large outdoor seating area. It’s also convenient to eat there for a quick lunch or dinner. I would definitely come back here again and try some of their other items.
0.4	I used to love this place, but when I moved to Austin, I wasn’t really sure what to expect. I started with a fish taco and a good sized selection of delicious seafood. The wait time was definitely worth it.I’d had my fish tacos before and all the people in the back were very accommodating. My friend had a plate of salmon tacos, and they were good. The tuna taco was perfect, and the fish was delicious and fresh. The chips were huge and the portions were huge. There were three different meats that I thought were excellent. The chips were super fresh and delicious. The chorizo tacos were okay, but the fish tacos were okay. The only thing I didn’t like was that there was a small side of fish and that my friend didn’t want to eat it. The fish tacos were pretty good too, but the price was a little too much for what I was getting. If I could give it 5 stars, I’d probably give it 5 stars.
0.5	I have been coming here for a year now. I have had a lot of bad experiences with the staff. One of the bad experiences was that the manager called the girl on the phone to tell her I was being watched. After being told that I was being watched, she ended up trying to tell me I was being watched. I was on the phone for a few minutes to talk with a manager at this time, who was not there. The manager never came back to check on me.After checking in with a manager, she was completely ignored. I had to call the manager to tell her about the problem. I have never had an issue with the staff at a McDonald’s. If the staff at a McDonald’s was really bad, why is the manager going to do anything to help them?
0.6	This place is just so amazing! I have been here a few times, but the first time I went it was just a small place with the only other person in the place. So the food was great, the service was fast, and the drinks were great. My friends and I were there at 7 pm on a Sunday (we were just there for a party) and were told it would be closed on the following Saturday. This made the wait even longer, and we were told it would be closed on a Saturday night. (This was done to accommodate our party’s schedule). The waitress was so nice and accommodating, and the food was delicious. The service was quick, but the food was very undercooked. I am not a big fan of chicken and it was like a chicken and egg dish, but this was the best I’ve had.I’d recommend this place if you are looking for a great meal to spend your weekend. I’ll definitely be back!
0.7	We have been to Oahu’s most beautiful Hawaiian restaurants in the past, and have always had great service. The wait staff is friendly, friendly, and have a great sense of humor. I like to try their drinks. The food is delicious. We’ve ordered a small number of their specials and have never had a bad experience. We have also ordered several of their “merchants” and have had great customer service. I can’t wait to come back to visit these other places in the area. I don’t know if they are running out of good things to say or just are trying to improve the overall experience.However, I know this place will stay busy for years to come. If you love Hawaiian food, consider coming here.
0.8	This is a bar that I have been to many times in the past, and I love it. I have been to a couple bars in the area before, and this is a different bar. I had been to the Ole Miss bar before, and this time it was better. The bartenders are very nice, and they have a nice setting, which is nice. There is a decent amount of people working, and the bartenders are all friendly and polite, which is a great thing. The food is delicious. I had the shrimp appetizer, and it was soooo good! I also had the pescatarian, which was a bit over cooked, but it was really good. The main courses were the chicken, salmon and tuna, and the salad. The salmon was so good, and I thought the dressing was a bit too oily. I could see it in the picture. The salad was a little on the salty side, but it was so good.I also had the steak and onion soup, which was pretty good. The dish was a little over cooked, but it was so good, it was almost like eating an egg on top of the spinach salad.The drinks were good, too. The food is pretty good, too.But, it’s just my opinion on this bar. I wouldn’t go back for that, but I will give it another shot!
0.9	I wish I could give 4 stars but I have to admit I was pleasantly surprised at how well the service was. The sushi was excellent and the hot springs were great. The wait staff was great as well. The server was very friendly and had a great sense of humor. I had the sweet potato shrimp with tomato sauce and a tuna fish salad. I also had the crab cake with corn bread. The crab cake was good but was definitely a bit too small for my liking. The salmon was a bit oily and I was disappointed. I can’t really say I would come back. The service was great. It was a great time. It took me about 15 min to get my drink, and after that I was able to get my drink from the counter. It was a nice little break and I didn’t feel rushed by the wait staff. The drinks were also nice. The service was very good. I would definitely come back.
1.0	So I walked in and it was a little boring. I like the fact that it’s a retail place. The staff was friendly and very welcoming, and I can say I had a good experience. The service was quick and friendly. The waiters are super friendly, friendly, and considerate. I had a little too much food and the bar seemed empty. I went on a Saturday night and the hostess came out and said she was going to be late, and that it was a bad night. I’m not sure why she’s not in there. I had a group of 20 people come in, and the hostess said the food was pretty good, but it wasn’t the best I’ve had. I really wanted to try this place but I ended up not being there.

**Table 7:** Conditional text generation.

**Title: The Infrared Astronomical Mission AKARI**
In this work we report on the IR Astronomical Mission AKARI (AKARI) based on the *Z*-critical stage for the optical observation of the Galactic dark energy payloads. The mission will be able to record and analyze the infrared and diffuse light from the gas clouds of the Galactic centre. MAGIC from the cosmic microwave background is anticipated to be several hundred times more powerful at deep wavelengths than the Hubble Space Telescope. We will be able to observe radiofrequency signals from the radio galaxies in the brightest regions of galaxies. We will be able to link the radio emission with the biological activity of the galaxy, thereby providing the closest scientific opportunity to examine the evolution of the Galactic Dark Energy.
**Title: Polarized States and Domain Walls in Spinor Bose-Einstein Condensates**
A representative dataset of spinor Bose-Einstein condensates (BSF) is investigated through a special set of independent finite fields. Switching of the field between spinor states and domain walls is considered in the sense that each spinor can be viewed as a spinor-pole. The corresponding degree of freedom is found to be in agreement with the classical relations, and the associated scaling function is modeled as a sum of two functions. It is shown that, for a given spinor, the resulting derivative of the momentum is in good agreement with the classical one. The temperature dependence, as well as a generalization of the thermodynamic stability, of the corresponding free energy are modeled in terms of spinors and their interactions. The results reveal a remarkable resemblance to experimental results, with the temperature dependence being nearly identical to those obtained by investigating spinor equivalence among spinors for finite length fields.

## Data Availability

Data sharing is not applicable for this article.
